# Andrographolide, A Natural Antioxidant: An Update

**DOI:** 10.3390/antiox8120571

**Published:** 2019-11-20

**Authors:** Eugenie Mussard, Annabelle Cesaro, Eric Lespessailles, Brigitte Legrain, Sabine Berteina-Raboin, Hechmi Toumi

**Affiliations:** 1 Laboratory I3MTO, EA 4708, Université d’Orléans, 45067 Orléans CEDEX 2, France; eugenie.mussard@univ-orleans.fr (E.M.); annabelle.cesaro@univ-orleans.fr (A.C.); eric.lespessailles@chr-orleans.fr (E.L.); 2 Service de Rhumatologie, Centre Hospitalier Régional d’Orléans 14 Avenue de l’Hôpital, 45100 Orléans, France; 3 Plateforme Recherche Innovation Médicale Mutualisée d’Orléans, Centre Hospitalier Régional d’Orléans 14 Avenue de l’Hôpital, 45100 Orléans, France; 4 NOVAXIA-6 Rue des champs Godin, 41220 St Laurent Nouan, France; b.legrain@labo-novaxia.com; 5 Institut de Chimie Organique et Analytique, Université d’Orléans-Pôle de Chimie, UMR CNRS 7311, Rue de Chartres, 45067 Orléans, France; sabine.berteina-raboin@univ-orleans.fr

**Keywords:** *Andrographis paniculata*, andrographolide, oxidative stress, Nrf2

## Abstract

Traditionally, *Andrographis paniculata* has been used as an herbal remedy for lung infection treatments. Its leaves contain a diterpenoid labdane called andrographolide responsible for a wide range of biological activities such as antioxidant, anti-inflammatory, and anti-cancer properties. This manuscript is a brief review of the antioxidant mechanisms and the regulation of the Nrf2 (nuclear factor (erythroid-derived 2)-like 2) signaling pathway by andrographolide.

## 1. Introduction

**Figure 1:**
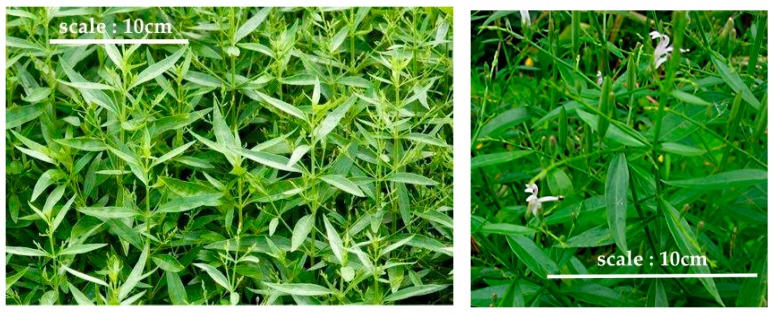
Andrographis paniculata.

**Figure 2:**
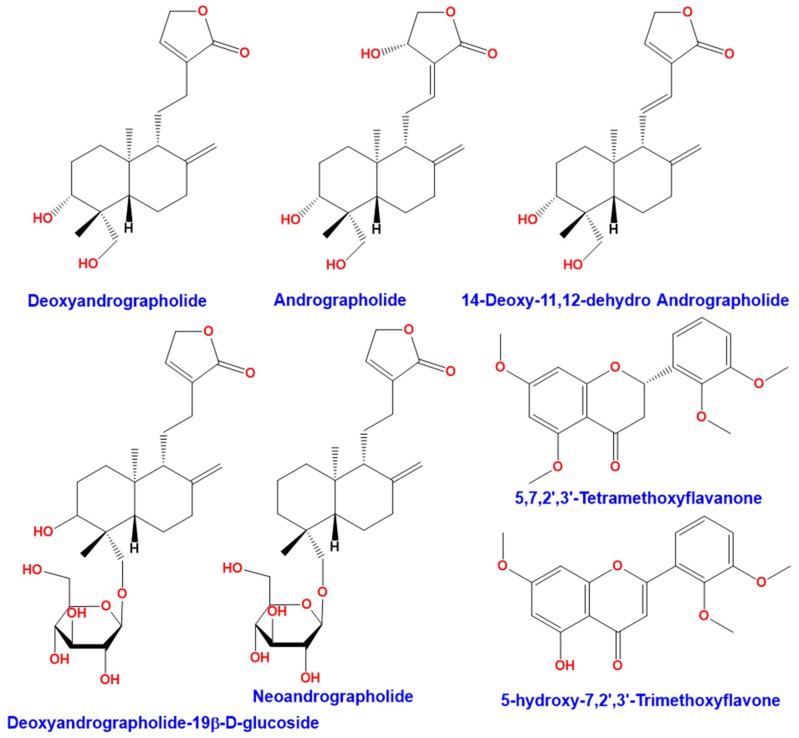
Compounds found in the leaves of *Andrographis paniculata.*

*Andrographis paniculata* (Burm. F.) Wall ex Nees is a plant from Acanthaceae family. This plant is also known as “chuan-xin-lian” in China, “kalmegh” in India, “senshinren” in Japan, “hempedu bumi” in Malaysia, “fah talai” in Thailand, and “green chiretta” in the Scandinavian countries [1]. *Andrographis paniculata* (Figure 1) has medicinal properties and has traditionally been used in India, Sri Lanka, China, and other countries in Southeast Asia [2]. This plant is widely recognized for its therapeutic properties against upper respiratory tract infections due to its high anti-inflammatory activity [3]. *Andrographis paniculata* has several biological activities. It has been used as an antipyretic [4,5], for hepatoprotective activity [6,7], and an immunostimulant [8,9]. The leaves of *Andrographis paniculata* contain many bioactive compounds including diterpene lactones (deoxyandrographolide, andrographolide, neoandrographolide, and 14-deoxy-11, 12-didehydroandrographolide), diterpene glucoside (deoxyandrographolide19β-d-glucoside), and flavonoids (5,7,2′,3′-tetramethoxyflavanone and 5-hydroxy-7,2′,3′-trimethoxyflavone), summarized in Figure 2 [10]. Discovered in 1951, andrographolide (C_20_H_30_O_5_) is the main active ingredient in the plant [11]. It is a lactone diterpene that gives the plant a bitter taste. Many studies have focused on the anti-viral [12,13,14], anti-thrombotic [15,16], hepatoprotective [17,18], anticancer [19,20], and anti-inflammatory properties [21] of andrographolide.

Several data have reported the antioxidant activities of andrographolide in various in vitro and in vivo model systems [22,23]. This review describes its pharmacokinetic parameters and examines the current status regarding the antioxidant effect of andrographolide.

The following antioxidant effects are explored: (1) reactive oxygen species (ROS) scavenging (2) mitochondria protection, (3) inhibition of ROS-producing enzymes, (4) antioxidant enzymes regulation, and (5) transcription factor Nrf2 (nuclear factor (erythroid-derived 2)-like 2) control.

## 2. Andrographolide Bioavailability

Most phytochemicals are poorly absorbed, rapidly metabolized, and excreted, resulting in low bioavailability [24]. Pharmacokinetics studies are important for understanding biological properties.

It has been reported that andrographolide is rapidly absorbed and metabolized in rats. A concentration of 1 µM (0.35 µg/mL) in plasma is obtained within 30 min after administration of 50 mg/kg and bioavailability is 1.19% [25]. Andrographolide was measured in plasma and various rat tissues after oral administration of andrographolide at a dose of 100 mg/kg/day for 4 weeks. The highest concentration of andrographolide was in the kidney, followed by the liver, spleen, and brain, while the same concentration was found in the heart and lungs. The maximum concentration was calculated at 115.81 ng/mL at 0.75 h. Andrographolide half-life elimination was 2.45 h [26]. Andrographolide was also measured in human plasma after an oral dose of 200 mg of andrographolide. The maximum concentration was calculated at 58.62 ng/mL at 1.6 h. Its elimination half-life was 10.50 h [27].

Andrographolide is insoluble in water and non-polar solvents but soluble in acetone, methanol, chloroform, and ether [28]. Therapeutic use of andrographolide is limited by its low water solubility, resulting in small bioavailability after oral administration. For this reason, the use of vectors such as microparticles or nanoparticles is interesting for its formulation for therapeutic applications. The microparticles used include polylactic-glycolic acid, alginic acid, and glucan derivatives. For nanoparticles, several nanocarriers are used, such as vesicles, polymeric nanoparticles, solid lipid nanoparticles, gold nanoparticles, nanocrystals, microemulsions and nanoemulsions, and nanosuspensions [29]. In order to increase the stability and bioavailability of andrographolide, solid lipid nanoparticles loaded with andrographolide have been prepared by a high-pressure homogenization method. Andrographolide bioavailability was obtained by the lipid combination (lecithin, glyceryl behenates, and glycerol monostearate) and the surfactant solution (3.0% Tween-80). A high pressure homogenization permitted the mixing and the stabilizing of the andrographolide with the other components. It also allowed andrographolide to be effective and furtive for the organism. It has been demonstrated that the bioavailability of andrographolide was increased to 241% by nanoparticles compared to the andrographolide suspension [30]. These methods avoid andrographolide insolubility in water.

## 3. Antioxidant Proprieties of Andrographolide

Andrographolide contributes to antioxidant defenses [31,32,33]. It acts directly by neutralizing free radicals. Also, it interferes indirectly by protecting mitochondrial integrity, inhibiting pro-oxidant enzymes, and/or activating antioxidant enzymes. Note that the transcription factor Nrf2 is involved in the regulation of the antioxidant defense system. Hence, the Nrf2 regulation by andrographolide is of interest for the regulation of the redox system.

### 3.1. Reactive Oxygen Species (ROS) Scavenging

ROS are oxygenated chemical species such as free radicals, oxygen ions, and peroxides. ROS have at least one unpaired electron, which makes them highly reactive. The following are some examples of ROS: singlet oxygen, hydrogen peroxide, and hydroxyl radical. They are continuously produced by ionizing radiation such as sunlight, as well as byproducts of cellular metabolism [34]. These compounds, when they are found in excess in the cells and go beyond the cellular defense systems, lead to a phenomenon called oxidative stress. They become toxic, and are important factors for many diseases such as diabetes [35], inflammation [36], and cancer [37,38].

A study has shown that the andrographolide exhibited significant antioxidative property (IC_50_: 3.2 µg/mL) by its ability to scavenge a stable free radical 1,1-diphenyl-2-picrylhydrazyl (DPPH) as compared to known antioxidants such as ascorbic acid, butylated hydroxy toluene (BHT), and the plant extract [39]. In fact, scavenging activity varied with concentration. It was up to a maximum of 40 mmol/mL for ascorbic acid and BHT, and 300 mg/mL for *Andrographis paniculata* extract and 15 mmol/mL for pure andrographolide. For ascorbic acid, BHT, *Andrographis paniculata* extract, and andrographolide, the IC_50_ values calculated were 4.3, 5.8, 220.5, and 3.2 mg/mL, respectively. Note that andrographolide had the highest antioxidant properties in this study, with the lowest IC_50_ value [39].

Several methods have been developed to determine andrographolide composition in leaf extracts. Different solvents are used at different extraction times. The amount of andrographolide in these extracts is important. One study used the HPLC-UV-MS method and the DPPH test, determining the free radical scavenging activities in the different extracts. The radical scavenging activity of all samples was lower than the positive control, BHT [40]. A study showed that an aqueous extract of *Andrographis paniculata* exhibited greater antioxidant activity than an ethanol extract. With 50 μg/mL, the radical scavenging activity was 66.8% in the aqueous extract versus 57.8% in the ethanol extract. These results are explained by a higher concentration of total flavonoids in the aqueous extract compared to ethanol extract. Flavonoids and phenols are known to be the main antioxidant compounds in plants. Interestingly, in this study, the ethanol extract contained more phenols than the aqueous extract; however, the aqueous extract was more potent than the ethanol extract in antioxidant activities [41].

In cellular models, andrographolide reduces the generation of ROS [42,43,44]. Indeed, in murine RAW264.7 macrophages, treatment with 10 and 30 μM of andrographolide reduced the production of ROS in these cells stimulated by Lipopolysaccharide (LPS) or Ovalbumin [42]. Sheeja et al. evaluated the antioxidants and anti-inflammatory properties of methanolic extract of *Andrographis paniculata*. This extract was found to inhibit the formation of oxygen-derived free radicals such as superoxide (32%), hydroxyl radicals (80%), lipid peroxidation (80%), and nitric oxide (42.8%) in an in vitro model. In vivo studies using mice models also showed significant inhibition in phorbol-12-merystate-13-acetate-induced superoxide (32.4%) and nitric oxide (65.3%) formation [43]. Shen et al. showed that andrographolide (0.1, 1, and 10 µM) inhibited intracellular ROS (singlet oxygen and hydrogen peroxide) in *N*-formyl-methionyl-leucyl-phenylalanine-induced neutrophils [44].

In *Andrographis paniculata* extracts, the ROS scavenging observed can be explained by the content of flavonoids and phenolic compounds [41,43]. However, ROS scavenging activity obtained from pure andrographolide is more surprising given its chemical structure [39].

### 3.2. Protective Effects of Andrographolide on Mitochondria

Mitochondria are organelles whose main function is to generate energy to the cells. Indeed, in the inner membrane, the electron transport chain generates ATP (adenosine triphosphate) from ADP (adenosine diphosphate). This process is called oxidative phosphorylation. The electrons are introduced into the complex I via NADPH and into the II complex via FADH2. Then, it is transferred to complex III and finally to complex IV. In the IV complex (cytochrome c oxidase), electrons are deposited in molecular oxygen, which leads to H_2_O production. However, electrons can be transferred to oxygen at complexes I and III to form superoxide (O_2_•^−^) rather than of H_2_O. This superoxide can damage macromolecules, such as, for example, DNA, proteins, or lipids.

Andrographolide improves mitochondrial dysfunctions in various models both in vivo and in vitro. Andrographolide treatment reduces oxidative stress and protects the mitochondria. These effects were observed in a transgenic mouse model (Amyloid precursor protein/presenilin 1) to imitate Alzheimer’s disease. Mice received andrographolide sulfonate at a dose of 5 mg/kg/day from two-month-old mice that lasted for 7 months. Mitochondria in the hippocampus of APP/PS1 mice were isolated. Treatment with andrographolide maintained the ATP content nearly to a normal level. It reduced oxidative stress and maintained the potential of the mitochondrial membrane. It also diminished mitochondrial swelling in APP/PS1 mice [45]. This confirms that andrographolide has a neuroprotective effect through its activity on mitochondria.

In another model, mitochondria were isolated from rat brains. Rats received nicotine (1 mg/kg/day) for 7 days and simultaneously andrographolide or an aqueous extract of *Andrographis paniculata* (250 mg/kg/day). Mitochondrial activity was tested on the following complexes: complex I (Dichlorophenol-indophenol-coenzyme Q reductase), complex II (succinate dehydrogenase coenzyme Q reductase), and complex III (coenzyme Q cytochrome c reductase). Measurements were performed in different regions of the brain—the cerebral hemisphere, the cerebellum, the diencephalon, and the brain stem. Supplementation with andrographolide or aqueous extract significantly increased the activity of mitochondrial complexes in the electron transport chain (I, II, III) and decreased the production of NO, malondialdehyde, and carbonyl protein in the rat brain exposed to nicotine. Aside from this, these treatments increased the activity of superoxide dismutase (SOD), catalase (CAT), glutathione reductase (GR), Glutathione peroxidase (GSH-Px), (Glutathione-S-transferase) GST, and reduced glutathione (GSH), as well as glutathione disulfite (GSSG) concentrations [46]. Andrographolide also acts in mitochondria of hepatic origin. Rats were exposed to copper (15 mg/kg/day) for 45 days and then treated with andrographolide at a dose of 20 mg/kg/day for 15 days. Andrographolide reduced the superoxide anions production and restored the membrane potential of mitochondria [47]. Andrographolide also inhibited LPS-induced superoxide production in mouse peritoneal macrophages. This inhibition was dose-dependent (0.1 to 100 μM) with an IC_50_ of 7.9 μM. Another compound from *Andrographis paniculata* was tested. It is called neoandrographolide, an andrographolide analogue. Neoandrographolide also suppressed LPS-induced NO production with an IC_50_ of 35.5 μM [48]. Note that andrographolide and an aqueous extract of *Andrographis paniculata* were also used in lymphocytes exposed to nicotine (100 µM). Results showed andrographolide or aqueous extract at 5, 10, and 20 µM reduced oxidative stress and decreased (1) superoxide anion production, (2) lipid peroxidation, (3) protein oxidation, and (4) DNA fragmentation. However, it increased cell viability, SOD, and GSH activity [49].

The energy production in the mitochondria is essential for cell survival. However, excessive production of mitochondrial ROS is harmful to cells. Andrographolide activity in mitochondria is beneficial for healthy cells and also anti-tumor activity in cancer cells via mitochondria. In fact, recent studies have shown that andrographolide causes cell death involving mitochondria in HeLa cells [50], a 5-FU colorectal cancer line [51], and finally liver cancer cells [52].

### 3.3. Inhibition of Free Radical-Producing Enzymes by Andrographolide

#### 3.3.1. NADPH Oxidase

NADPH oxidase is a membrane enzyme complex belonging to the class of oxidoreductases. This enzyme catalyzes the oxidation reaction of NADPH by oxygen (O_2_) to NADP^+^, H^+^, and O_2_•^−^. NADPH oxidase induces the production of ROS in cells. In humans, there are seven NADPH oxidase (NOX) types: NOX1, NOX2, NOX3, NOX4, NOX5, DUOX1, and DUOX2. It is composed of six functional subunits. Cytochrome b558 is integrated into the membrane. It consists of two subunits: p22^phox^ and NOX2. The G protein called Rap 1A binds the guanosine triphosphate/guanosine diphosphate (GTP/GDP). It is linked to cytochrome b558 and is activated when associated with GTP. The p47^phox^ subunit acts as an organizer by translocating the p67^phox^ and p40^phox^ subunits to the membrane. The p67^phox^ subunit associates with cytochrome b558 to activate the oxidase activity of the enzyme. The p40^phox^ subunit plays a role in regulating the enzymatic activity. Finally, the Rac subunit is cytosolic and linked to the GDP in its non-activated form. When activated, it binds to GTP and associates with cytochrome b558 on the membrane.

Andrographolide (100 μg/kg, iv) reduced brain damage in a mouse model of cerebral ischemia. It reduced NOX2 (gp91^phox^) expression via a limiting of Phosphoinositide 3-kinase/ Protein Kinase B (PI3K/AKT)-dependent nuclear factor-kappa B (NF-κB) activation. Andrographolide (10 µM) reduced NOX2 expression in BV2 microglial line after 8 h of oxygen-glucose deprivation [53]. In a diabetic mouse model induced by intraperitoneal injection of streptozotocin, the effects of andrographolide on the myocardium were explored. Results demonstrated that andrographolide improved the deleterious effects of oxidative stress by NADPH oxidase reduction. Indeed, andrographolide (10 and 20 mg/kg/day) significantly decreased of NOX2, NOX4, and p47^phox^ expressions in the myocardial tissues. Similarly, the effect of andrographolide was shown in H9c2 cardiomyoblasts exposed to high glucose (25 mM). Treatment with andrographolide (1, 5, and 10 µM) significantly reduced the expression of NOX2, NOX4, and p47^phox^ [54]. It modified the NADPH oxidase expression but it also modified its activity. It inhibited the NADPH oxidase activation in the lung tissue of rats after treatment with LPS (5 mg/kg). Andrographolide (0.18 and 1.8 g/kg) also decreased the translocation of the p47^phox^ and p67^phox^ subunits from the cytoplasm to the nucleus [55]. To activate NADPH oxidase, tumor necrosis factor-alpha (TNF-α) prompts the translocation of the p47^phox^ and p67^phox^ subunits from the cytoplasm to the nucleus. In the endothelial line EA.hy926, andrographolide (7.5 µM) reduced the translocation of the p47^phox^ and p67^phox^ subunits [56]. In human colorectal cancer line HCT116 cells, andrographolide (20 µM) decreased the translocation of the p47^phox^ subunit [57].

Oxidative stress is involved in many diseases [58]. However, the production of low ROS concentration acts as messengers to activate signaling pathways. Therefore, the generation of ROS in low concentration is of interest. ROS production activates the antioxidant system in order to anticipate and protect against oxidative damage. For example, this “vaccine” effect has been studied in RIN-mβ cell line of the pancreas with an andrographolide derivative containing a lipoic acid. Andrographolide-lipoic acid conjugate (0.01–0.1 and 1 µM) increased the ROS level in a dose-dependent manner after 1 h by increased expression of NADPH oxidase. As a result, the expressions of the antioxidant proteins Thioredoxin-1 (Trx1), Peroxiredoxin-1 (Prx1), Peroxiredoxin-5 (Prx5), Heme oxygenase-1 (HO-1), SOD1, and SOD2 were up-regulated. Together, they protect the cells from H_2_O_2_-induced apoptosis [59].

#### 3.3.2. Xanthine Oxidase

Xanthine oxidase catalyzes the terminal steps of purine degradation, converting hypoxanthine to xanthine, and xanthine to uric acid and hydrogen peroxide. In humans, xanthine oxidase controls the final step of purine catabolism and is normally found in the liver and the intestinal mucosa. Xanthine oxidase is considered an essential source of O_2_•^−^ and H_2_O_2_ in inflammatory diseases [60,61].

Currently, no study has proven any effect of pure andrographolide for xanthine oxidase. However, a single report from Lin et al. showed an inhibitory effect to xanthine oxidase activity by two extracts of *Andrographis paniculata*. The extracts were obtained from water or ethanol extraction. The aqueous extract and ethanolic extract inhibited xanthine oxidase activity with an IC_50_ of 13.62 µg/mL and 26.60 µg/mL, respectively [41]. Both extracts contained andrographolide. Nevertheless, in this study, there was no evidence that the observed effect came from andrographolide. However, a silico study showed strong binding interactions between xanthine oxidase and andrographolide. The interaction was with four amino acids and the binding energy was −4.57 kcal/mol [62]. These results suggest that andrographolide may be a potent inhibitor of xanthine oxidase.

### 3.4. Antioxidant Protective Proprieties of Andrographolide in Activation of Antioxidant System

Maintaining a non-cytotoxic level of ROS is provided by endogenous and exogenous antioxidant systems. There are two sources of antioxidants: one provided by the diet, whereas the other is internal and consists of non-enzymatic or enzymatic cellular antioxidants [34]. Here, we focus on superoxide dismutase (SOD), catalase (CAT), and glutathione peroxidase (GPx).

#### 3.4.1. SOD, CAT, and GPx

SOD represents the first defenses of the body against oxidative stress. SOD ensures the elimination of the superoxide anion in hydrogen peroxide by a dismutation reaction (2O_2_^−^ + 2H^+^ → H_2_O_2_ + O_2_). Then, H_2_O_2_ is supported by enzymes with peroxidase activity. In mammals, there are three isoenzymes: a cytosolic and nuclear form associated with copper and zinc ions (CU/ZN-SOD_1_), a mitochondrial form associated with manganese (Mn-SOD_2_), and an extracellular form (EC-SOD_3_). These enzymes differ in their chromosomal location, quaternary structure, metal content, and cell localization [63].

Catalase catalyzes the disproportionation of hydrogen peroxide into water and oxygen (2H_2_O_2_ → 2H_2_O + O_2_). CAT is mainly located in the peroxisome, and also in the cytoplasm. CAT protects against the harmful effects of excessive amounts of H_2_O_2_ [64].

GPx is a complete system that reduces peroxides at the expense of its specific substrate, reduced glutathione (GSH). GPx plays a central role in the H_2_O_2_ removal mechanism. Its main role is lipid peroxide elimination resulting from oxidative stress action on polyunsaturated lipids. The GPx-catalyzed reaction requires the presence of GSH as the electron donor (2GSH + H_2_O_2_ → GSSG + 2H_2_O). The product formed is glutathione disulfite (GSSG). GSSG is then reduced by glutathione reductase (GR) using NADPH (GSSG + NADPH + H^+^ → 2GSH + NADP^+^) [65,66]. The GSH/GSSG ratio is an index of the oxidation state in the cells [67]. There are several isoforms of GPx containing selenium: cytosolic and mitochondrial GPx, cytosolic GPX, and extracellular GPx.

#### 3.4.2. In Vivo Studies

Several studies have shown that andrographolide restores SOD and CAT activities in cells treated with oxidative stress inducers. For example, oral treatment with andrographolide (5 mg/kg, 7 mg/kg and 10 mg/kg) restored SOD and CAT activities due to treatment with hexachlorocyclohexane [68]. In rat erythrocytes, SOD and CAT activities were reduced by treatment with Carbon tetrachloride (CCl_4_). Treatment of rats with a methanolic extract of *Andrographis paniculata* (1 g/kg) restored SOD and CAT activities after stimulation by CCl_4_ [69]. Another dataset reported that SOD activity was significantly increased in serum of hyperglipidemic rats by oral administration of andrographolide 10 and 20 mg/kg [70]. SOD activity increased in the liver, kidneys, heart, and red blood cells of rats treated with andrographolide (30 and 50 mg/kg/day). Catalase activity increased in a dose-dependent manner only in the heart of treated rats. SOD1 protein levels in the liver, kidneys, and heart of rats were increased by treatment with andrographolide. Finally, SOD1 mRNA levels increased in the liver and kidneys of rats treated with andrographolide [25]. A study on atherogenic rabbits showed that the bacterium *Porphyromonas gingivalis* decreased the activities of SOD and CAT. These effects were reversed by oral administration of andrographolide at 10 or 20 mg/kg [71]. SOD, CAT, and GSH activities decreased in the gastric tissue of rats treated with indomethacin. Oral administration of andrographolide sodium bisulfate (40, 80, and 160 mg/kg) 7 days before treatment with indomethacin reduced oxidative stress by restoring SOD, CAT, and GSH activities [72]. Mice treated with arsenic have decreased SOD and CAT activities. Oral administration of andrographolide or andrographolide nanoparticles increased these activities in groups co-treated with arsenic [73]. SOD and CAT activities are decreased in the brains of diabetic rats. These reductions were less significant in diabetic mice treated orally with andrographolide at doses of 15, 30, or 60 mg/kg or with hydromethanolic extract of *Andrographis paniculata* [74]. Another diabetes study showed similar results in a model of rats with diabetes mellitus by streptozotocin injection. SOD and CAT activities were reduced in the hippocampus, hypothalamus, and cerebellum of the cerebral cortex of the rat brain. However, andrographolide supplementation (2.5 mg/kg) reduces SOD and CAT activities to normal levels [75]. In the lung tissues of mice treated with bleomycin, SOD activity decreased compared to untreated mice. Co-treatment with andrographolide (25, 50, and 100 mg/kg) significantly increased the SOD activity in a dose-dependent manner [76]. Topical application of sodium bisulfate andrographolide (0.4–1.2 and 3.6 mg/mouse) to the skin of mice exposed to UV radiation resulted in a dose-dependent increase in SOD and CAT activity compared to untreated mice [77]. In contrast, andrographolide sodium bisulfate significantly decreased SOD activity in the kidneys of mice treated with 150 and 1000 mg/kg [78].

#### 3.4.3. In Vitro Studies

Some studies show the effect of andrographolide on antioxidant enzyme activity in vitro models. In RIN-m lineage, andrographolide conjugated with alpha-lipoic acid increased SOD and CAT activities [79]. Similarly, in chondrocytes isolated from rat articular cartilage, andrographolide at 0.625, and 2.5 µg/mL increased SOD protein content; SOD and CAT activity; and SOD1, SOD2, and CAT expression after exposure with H_2_O_2_ [31]. In contrast, in HK-2 human kidney cell line, treatment with andrographolide sodium bisulfate decreased SOD activity by 30 to 120 µM in a dose-dependent manner, and significantly from 60 µM onwards [80]. This decrease contributes to the induction of cell apoptosis by oxidative stress. These results are supported by another study on lymphocytes isolated from rats. In this report, SOD activity was reduced in the presence of nicotine. Treatment with andrographolide (5, 10, and 20 µg/mL) or an aqueous extract of *Andrographis paniculata* improved SOD activity in lymphocytes [49].

### 3.5. Nrf2 Signaling Pathway: Andrographolide Regulation

Nrf2 (nuclear factor (erythroid-derived 2)-like 2) is an essential transcription factor against oxidative and electrophilic stress. There is clear evidence that it plays a key role in balancing oxidation-reduction reactions by activating a wide variety of genes involved in antioxidant defense. This protective role consists in inducing the expression of specific enzymes. These are antioxidant enzymes, phase II enzymes, and detoxification enzymes. These enzymes contain ARE (antioxidant response element) and EpRE (electrophilic response element) DNA sequences on their promoters. The Nrf2 targets are therefore ROS chelating enzymes, detoxification enzymes, or phase II enzymes. Several studies have identified genes regulated by Nrf2, such as those involved in glutathione biosynthesis as gamma-glutamylcysteine synthetase (*γ-GCS*), glutamate cysteine ligase (*GCL*), glutathione reductase (*GR*), glucose-6-phosphate dehydrogenase (*G6PDH*), *GST*, and *GPx*), the NAD(P)H dehydrogenase [quinone] 1 (*NQO1*), *HO-1*, *SOD*, *CAT*, and thioredoxin reductase (*TRXR*) genes.

In physiological condition, Nrf2 is maintained in the cytoplasm by forming an inactive complex with the Keap-1 protein (Kelch-like ECH-associated protein 1). Keap-1 is anchored to the cytoskeleton via actin. The binding between Nrf2 and Keap-1 facilitates the ubiquitination and proteolysis of Nrf2 via the Cul3-based E3 complex. Keap-1 is an essential regulator of the antioxidant response because it captures oxidation-reduction changes by some of its cysteines. Its N-terminal domain contains the residue Cys^151^, which is important for the detection of oxidative stress. Its IVR (intervening region) domain contains the residues Cys^273^ and Cys^288^, which are also involved in the detection of oxidative stress. Its DGR (double glycine repeat) domain and its C-terminal domain form a structure to interact with Nrf2. Phosphorylation of Nrf2 at its serine and/or tyrosine residues also causes the release of Nrf2. Therefore, its activity can be increased via protein stabilization by Keap-1 modifications or Nrf2 phosphorylation.

#### 3.5.1. In Vitro Studies

Andrographolide induces an increase in Nrf2 expression and its translocation to the cell nucleus, independently of the cell type studied. This translocation increases ARE promoter and SOD, CAT, glutamate-Cysteine Ligase Catalytic Subunit (GCLC), glutamate-cysteine ligase modifier subunit (GCLM), sulfiredoxin-1 (SRXN1), thioredoxin reductase 1 (TXNRD1), glutathione-disulfide reductase (GSR), and glutathione reductase (GR) expressions. These enzymes have cytoprotective, antioxidant, and detoxifying effects. Also, the stress protein HO-1 is protective against oxidative aggressions. Its expression was increased by andrographolide via Nrf2. HO-1 generates antioxidant compounds including carbon monoxide, bilirubin, and free iron. In the absence of oxidative stress, Nrf2 remains sequestered in the cytoplasm by the Keap-1 protein, and it is rapidly degraded by the proteasome. In most studies, andrographolide does not appear to regulate Keap-1. The main effects of andrographolide on in vitro models are summarized in Table 1.

Andrographolide (2.5–5 and 7.5 µM) induces a dose-dependent increase in HO-1 expression in the endothelial cell line EA.hy926 after a 16 h pre-treatment followed by incubation with TNF-α (1 ng/mL) for 6 h. Treatment with 7.5 µM andrographolide improves the dissociation of Nrf2 to Keap-1 and their nuclear translocation. Nrf2 can bind to ARE sequences, which explains the increase in HO-1 expression in cells [81]. In another study on EA.hy926, treatment with 7.5 µM andrographolide induced HO-1 synthesis in these cells [82]. In the bronchial cell line BEAS-2B simulated by cigarette smoke extract, treatment with 30 µM andrographolide reduced oxidative stress. This treatment favored the nuclear translocation of Nrf2 and its binding to ARE sequences. This phenomenon thus led to positive regulation of the antioxidant genes *GCLM*, *GCLC*, *GPx-2*, *GR*, and *HO-1*. Cellular GSH levels were significantly increased by andrographolide in cells exposed to cigarette smoke extract for 24 h [32,83]. The human hepatoma line Huh-7 has decreased HO-1 promoter activity when it contains hepatitis C virus replicons (Ava5 cells). This activity increased in a dose-dependent manner when Ava5 cells were treated with andrographolide (1–5 and 10 µM) for 72 h. In correlation, HO-1 expression was dose-dependently increased with andrographolide (5, 7.5, and 10 µM). Finally, HO-1 protein synthesis increased significantly after 7.5 µM of andrographolide. The total amount of Nrf2 protein increased dose-dependently from andrographolide to 5 µM. The amount of nuclear Nrf2 protein also increased strongly from andrographolide to 7.5 µM. With 10 µM of andrographolide, the accumulation of Nrf2 protein in the nucleus increased with time, from 3 to 24 h of treatment. The DNA binding activity of Nrf2 decreased in Ava5 cells compared to Huh-7 control cells. Treatment with andrographolide (5, 7.5, and 10 µM) for 72 h significantly and dose-dependently increased this activity as early as 5 µM. No significant changes in Keap-1 protein levels were observed in the presence of andrographolide. The formed Nrf2 protein is therefore not advantageously eliminated by ubiquitination [84]. GSH content increased in the endothelial cell line EA.hy926 by treatment with andrographolide at 7.5 µM after 24 h. Only GCLM and HO-1 but no GCLC expressions increased time-dependently with treatment with andrographolide at 7.5 µM. Nrf2 was activated by andrographolide and participates in the induction of HO-1 and GCLM expression from 1 h of treatment and maintained up to 4 h [56]. In primary endothelial cells of mouse brains treated with andrographolide at 5 or 10 µM, the amount of HO-1 mRNA and protein increased with time. The increase in HO-1 protein was significant after 4 h of treatment and the increase in HO-1 expression was significant after 2 h of treatment with andrographolide at 10 µM. These inductions were greater with 10 µM andrographolide than with 5 µM. Andrographolide (10 µM) activates Nrf2 via its phosphorylation on ser^40^. A translocation of Nrf2 from the cytoplasm to the nucleus was also observed in cells treated with andrographolide (10 µM) after 30 min of treatment [85]. Primary culture of rat astrocytes treated for 1h with different concentrations of andrographolide showed an increase in Nrf2 protein. Treatment with andrographolide at 50 µM increased the RNAm Nrf2 from 24 h of treatment while the protein level increased significantly from 30 min of treatment and was maintained until 24 h. The positive regulation of protein level is therefore not related to the increase in gene expression but rather to the regulation of protein turnover, such as the improvement of protein stability. The Nrf2 protein increased significantly in the cell fraction and the nuclear fraction after 1 h and 30 min of treatment with andrographolide at 50 µM, respectively. However, andrographolide did not affect the phosphorylation of Nrf2 on ser^40^ or Keap-1 levels. Andrographolide, therefore, does appear to induce accumulation of Nrf2 in the nucleus via the escape of Nrf2 to its degradation by the proteasome. The cycloheximide use, an inhibitor of the initiation and elongation of de novo protein synthesis, showed that Nrf2 had a half-life of 10 min. However, with andrographolide (50 µM), its half-life was reduced to 40 min. Indeed, andrographolide reduced the ubiquitination of Nrf2. This mechanism can explain the increased stability of Nrf2 in cells and therefore the positive regulation of its effector genes. HO-1 expression was increased after 2 h of incubation with andrographolide at 50 µM, whereas the HO-1 protein level was increased after 4 h [86]. In HT22, a mice neuronal cell line, andrographolide increased cytoplasmic and nuclear protein levels in a dose-dependent manner after 24 h of treatment. These increases were significant at 10 µM. Also, the Keap-1 content was not modified by andrographolide at 10 µM for 24 h. The transcription activity of ARE sequences increased with andrographolide treatment (1, 5, and 10 µM) in a concentration-dependent manner for 16 h. HO-1 expression and HO-1 protein content increased and varied with andrographolide concentration for 24 h [87]. In H9c2, a rat myoblast cell line, stimulation with 25 mM glucose reduced the amount of Nrf2 and HO-1 proteins. Co-stimulation with andrographolide (0.1, 1, 5, and 10 µM) increased the Nrf2 and HO-1 proteins in the cells [54]. In chondrocytes isolated from rat articular cartilage, andrographolide at 0.625 and 2.5 µg/mL increased the Nrf2 protein after exposure with H_2_O_2_ [31]. The amyloid peptide beta 1-42 (Aβ42) (10 µM) reduced Nrf2 mRNA and protein after 24 h of treatment in the PC12 line derived from tumor cells of the adrenal medulla in rats. Pre-treatment with andrographolide at 20 µM for 1 h restored the Nrf2 protein content and increased its expression [88]. Andrographolide increased Nrf2 transcription activity and protein concentration in HEK293T cells. Indeed, the cells were treated with different concentrations of andrographolide (1, 7.5, 15, 30, 60 and 120 µM) for 4 h. The Nrf2 protein increased from 7.5 µM and in a dose-dependent manner up to 120 µM. In addition, andrographolide (7.5 µM) increased Nrf2 protein levels in a time-dependent manner from 1 to 8 h of treatment. The transcriptional activity of the ARE sequences was increased via treatment with andrographolide at 7.5 µM for 24 h. Andrographolide (7.5 µM) induced Nrf2 by regulating its Keap-1 inhibitor via interaction with Cys^151^ after 6 h of treatment [89]. This mechanism is similar to sulforaphane [90,91,92]. Andrographolide (7.5 µM) also caused a 30% decrease in the binding between CUL3 and Keap-1 depending on Cys^151^. This disruption had the consequence of inhibiting the transfer of ubiquitin to Nrf2 and therefore its degradation by the proteasome. At low concentration (7.5 µM), andrographolide decreased the binding between CUL3 and Keap-1 and stabilized the Nrf2 protein depending on Cys^151^, whereas treatment with a high concentration of andrographolide (100 µM) increased the binding from CUL3 to Keap-1 and induced Nrf2 independently of Cys^151^ [89]. *Andrographis paniculata* extracts enriched with andrographolide by phytoconcentration were studied. Effects on the Nrf2 pathway in a human hepatic cell line, HepG2, were observed [93]. An unenriched plant extract (AP), 10% (AP10), and 20% (AP20) enriched extracts of andrographolide and pure andrographolide at 20 µM (AN20) and 40 µM (AN40) were used. Except for the AP treatment, all treatments significantly increased Nrf2 expression. An increase total Nrf2 and nuclear Nrf2 was induced by all treatments. Moreover, all treatments except AP significantly increased HO-1 expression. Similarly, all treatments increased the HO-1 protein in the cells. HO-1 is positively regulated by Nrf2 and negatively by BACH-1 and mir-377. Andrographolide did not modify the BACH-1 expression. Yet, all treatments except AP had reduced mir-377 expression. The GSH content was increased by all treatments. In addition, GCLC, GCLM, and GS expressions were significantly increased by all treatments, particularly by AP20, by negatively regulating mir-433. Treatments increased GR mRNA, protein, and enzyme activity. On the other hand, GPx1 expression and total GPx activity were decreased by upregulating mir-181a [93].

New andrographolide derivatives have been discovered, some of which are natural derivatives, others of which are synthetic derivatives derived from chemistry [1,28,94]. For example, CHP1002 is a restructured derivative of andrographolide. This synthetic molecule has the same central nucleus as andrographolide and two polyethylene glycol molecules to improve water solubility and stability of andrographolide. In the RAW264 macrophage line, CHP1002 at 25–50 or 100 µM improved HO-1 protein levels in a dose-dependent manner. The HO-1 protein was increased from 4 h of treatment with a peak at 6 h. Moreover, the expression of the HO-1 gene was induced by CHP1002 after 2 h of treatment. The Nrf2 pathway was studied to elucidate the underlying mechanisms of HO-1 induction by CHP1002. Treatment with 100 µM of CHP1002 increased the level of Nrf2 protein in total cellular homogenates from 2 h after treatment to 8 h compared to the untreated control. Also, an accumulation of the Nrf2 protein in the nucleus was observed as early as 2 h after treatment, and persisted and increased until 6 h [95].

#### 3.5.2. In Vivo Studies

The main effects of andrographolide on in vivo models are summarized in Table 2. Andrographolide increased Nrf2 translocation and its binding activity to ARE sequences in the liver of Sprague–Dawley rats intragastrically treated with 30 or 50 mg/kg/day andrographolide for 5 consecutive days [25]. With regard to Nrf2 targets, the andrographolide treatment induced the following results:

- SOD activity increased in the liver, kidney, heart, and red blood cells;
- CAT activity increased in the heart;
- GSH peroxidase activity increased in the kidney;
- GSH reductase increased in the kidney, heart, and red blood cells;
- GSH S-transferase increased in the liver;
- GSH protein increased in the heart;
- antioxidant proteins (SOD1, GST Ya, GST Yb, HO-1, GCLC, and GCLM) increased in the liver, kidney, and heart;
- The mRNA of GCLC, GCLM, GST Ya/Yb, SOD1, and HO-1 increased in the liver and kidney.

Andrographolide increased HO-1 protein in the brain of Wistar rats treated with 0.1 mg/kg by intraperitoneal for 6 h [85]. It increased Nrf2 translocation and decreased Keap-1 mRNA expression in lung of BALB/c mice treated with 5 or 10 mg/kg andrographolide by intraperitoneal for 1 and 24 h [96]. In this study, mRNA expression of HO-1, GR, GCLM, GPx-2, and NQO1 increased by andrographolide treatment. Andrographolide increased Nrf2 and HO-1 proteins in liver of BALB/c mice treated with LPS/D-galactosamine (1 h) followed andrographolide (2.5, 5, or 10 mg/kg) by intraperitoneal for 8 h [6]. Andrographolide increased Nrf2 translocation in liver of C57BL/6 mice orally treated with acetaminophen for 2 weeks and co-treated with andrographolide (20 or 40 mg/kg) every day during an additional 4 weeks [33]. Moreover, andrographolide reversed the decreased hepatic expression of GCLC, GCLM, and HO-1 mRNA expression induced by acetaminophen. It increased Nrf2 mRNA in the heart of C57BL/6 mice treated with streptozotocin by intraperitoneal injection followed by intragastric gavage of andrographolide (1, 10, or 20 mg/kg/day) for 12 weeks [54]. In the same way, this treatment increased SOD activity, decreased malondialdehyde (MDA), and decreased mRNA of Nox2, Nox-4, p47^phox^, Nrf2, and HO-1.

Andrographolide increased Nrf2 protein in lung of Balb/c mice sensitized (dermally and intranasally) with toluene diisocyanate for asthma induction and treated with andrographolide 0.1, 0.5, or 1 mg/kg [97]. HO-1 protein increased only with the dose 1 mg/kg of andrographolide. A synthetic derivative of andrographolide by conjugation with an alpha-lipoic acid increased Nrf2 and HO-1 expression in the β cells of the Langerhans islets of rats RIN-m [79].

Overall, andrographolide improved Nrf2 expression, increasing its mRNA and protein. Andrographolide activated the Nrf2 pathway by increasing in its nuclear translocation. However, this mechanism does not appear to be related to the decreased expression of its inhibitor, Keap-1. Many studies showed the binding of Nrf2 to the ARE sequence, promoted by andrographolide. Nrf2 is considered to be the main regulator of oxidative stress. This transcription factor activates the expression of a wide variety of genes containing the ARE sequence in their promoter regions. These genes are involved in antioxidant cell defense. It includes antioxidant enzymes (SOD, CAT, and GPx), as well as detoxification enzymes (HO-1, NQO1, and GST), and other stress response proteins contributing to the fight against oxidative damage (γ-GCS, GR, GCLC, GCLM, G6PDH, and GR).

## 4. Conclusions

Medicinal herbs contain a wide range of active ingredients including antioxidants. The natural antioxidants are of interest for treating a large number of diseases due to oxidative stress. Moreover, *Andrographis paniculata* has long been used in traditional medicine in Asia. Several studies have shown that its main bioactive component, andrographolide, has shown beneficial effects against oxidative stress, notably through Nrf2 activation. To examine the various mechanisms involved, this review intended to collect wide range of studies focusing on the antioxidant activity of andrographolide.

In conclusion, there are several potential mechanisms to explain the antioxidant activity of andrographolide. These mechanisms can be direct [39] or indirect [93,97]. Andrographolide can prevent free-radical formation by protecting mitochondria or by inhibition of specific ROS-producing enzymes. It can also activate enzymatic or non-enzymatic antioxidants, mainly via the activation of the Nrf2 signaling pathway. Therefore, andrographolide use as an active ingredient is a promising strategy for the development of new anti-oxidant drugs.

## Figures and Tables

**Table 1:** Main effects of andrographolide on Nrf2 (nuclear factor (erythroid-derived 2)-like 2) pathway in vitro. Symbols: “↗” for an increase, “↘” for a decrease, and “-” for not communicated. ARE: antioxidant response element, Keap-1: Kelch-like ECH-associated protein 1, GSH: reduced glutathione, NOX: NADPH oxidase, SOD: superoxide dismutase, CAT: catalase.

Treatment(s)	Nrf2 mRNA	Nrf2 Protein	Nrf2 Translocation	Nrf2 Phosphorylation	Nrf2 Binding Activity to ARE Sequence	Nrf2 Inhibitor (Keap-1)	Nrf2 Turnover and Ubiquitination	Nrf2 Targets	Ref.
2.5, 5, and 7.5 µM for 16 h followed by incubation with TNF-α (1 ng/mL) for an additional 6 h	-	-	↗	-	↗	-	-	HO-1 mRNA and protein ↗	[81]
7.5 µM andrographolide for 16 h	-	-	-	-	-	-	-	HO-1 protein ↗	[82]
30 µM andrographolide with 2% cigarette smoke extract	-	-	↗	-	↗	-	-	GSH ↗; expression of antioxidants GCLM, GCLC, GR, GPx-2, and HO-1 ↗	[83]
30 µM for 24 h	-	-	↗	-	↗	-	-	↗ ARE-sensitive genes including HO-1, GCLC, GCLM, SRXN1, TXNRD1, and GSR but not NQ01	[32]
1, 5, 7.5, and 10 µM	-	↗	↗	-	↗	No effect	-	HO-1 promoter activity and mRNA ↗	[84]
7.5 µM andrographolide for 24 h	-	-	↗	-	-	-	-	HO-1 mRNA and protein ↗; GCLM mRNA and protein ↗ but not GCLC	[56]
5 and 10 µM	-	-	↗	p-Nrf2 (ser^40^) ↗	-	-	-	HO-1 mRNA and protein ↗	[85]
1, 5, 10, 30, and 50 µM	50 µM for 24h ↗	1–50 µM for 1h ↗, and 50 µM for 30 min to 24 h ↗	50 µM for 30 min to 24 h ↗	no effect on p-Nrf2 (ser^40^)	-	No effect	↘	HO-1 mRNA (2 h with 50 µM) and protein (4 h with 50 µM) ↗	[86]
1, 5, and 10 µM	-	↗	↗	-	↗	No effect	-	HO-1 mRNA and protein ↗	[87]
**Glucose 25 mM and andrographolide 0.1, 1, 5, and 10 µM**	-	↗	-	-	-	-	-	HO-1 protein ↗, Nox2, Nox4, and P47^phox^ mRNA ↘	[54]
H_2_O_2_ 0.1 mmol/L and andrographolide 0.625 and 2.5 µg/mL	-	↗	-	-	-	-	-	Activities of SOD and CAT ↗; SOD and CAT proteins ↗	[31]
β-amyloid (Aβ) peptide 10 µM and andrographoldie 20 µM for 1 h	↗	↗	-	-	-	-	-	-	[88]
1, 7.5, 15, 30, 60, and 120 µM	-	↗	-	-	↗	At low concentration, function of the CUL3-RBX1-KEAP-1 E3 ubiquitin ligase via Cys^151^ ↘ at high concentration action via a mechanism independent of Cys^151^ in KEAP-1	↗	-	[89]
20 and 40 µM	↗	↗	↗	-	-	-	-	HO-1 protein ↗; GSH protein ↗; GCLC/GCLM/GS mRNAs and proteins ↗; GR mRNA, protein, and activity ↗ but GPx1 mRNA and total GPx activity ↘	[93]

**Table 2:** Main effects of andrographolide on Nrf2 pathway in vivo, Symbols: “↗” for an increase, “↘” for a decrease, and “-” for not communicated.

Model(s)	Treatment(s)	Nrf2 mRNA	Nrf2 Protein	Nrf2 Translocation	Nrf2 Binding Activity to ARE Sequence	Nrf2 Inhibitor (Keap-1)	Nrf2 Targets	Ref.
Sprague–Dawley rats (liver, heart, and kidneys)	30 or 50 mg/kg/day for 5 consecutive days, intragastrical	-	-	↗ (liver)	↗ (liver)	-	SOD activity ↗ (liver, kidney, heart, and red blood cells); CAT activity ↗ (heart); GSH peroxidase activity ↗ (kidney); GSH reductase ↗ (kidney, heart, and red blood cells); GSH S-transferase ↗ (liver); GSH protein ↗ (heart); antioxidant proteins (SOD1, GST Ya, GST Yb, HO-1, GCLC, and GCLM) ↗ (liver, kidney, and heart); mRNA (GCLC, GCLM, GST Ya/Yb, SOD1, and HO-1) ↗ (liver and kidney)	[25]
Wistar rats	0.1 mg/kg, intraperitoneal (6 h)	-	-	-	-	-	HO-1 protein ↗ (brains)	[85]
BALB/c mice	5 or 10 mg/kg, intraperitoneal (1 and 24 h)	-	-	↗ (lung)	-	mRNA ↘ (lung)	HO-1, GR, GCLM, GPx-2, and NQO1 (mRNA) ↗	[96]
BALB/c mice	LPS/GalN (1 h) followed by andrographolide (2.5, 5, or 10 mg/kg), intraperitoneal (8 h)	-	↗ (liver)	-	-	-	HO-1 protein ↗ (liver)	[6]
C57BL/6 mice	Acetaminophen (orally) every day for 6 weeks followed by andrographolide (20 or 40 mg/kg, orally) treatment every day at 2 weeks after acetaminophen administration	-	-	↗ (liver)	-	-	ANDRO reversed the decreased hepatic expression of GCLC, GCLM and HO-1 mRNA expression induced by acetaminophen. Co-treatment with ANDRO (40 mg/kg) ↗ NQO1 mRNA	[33]
C57BL/6 mice	Streptozotocin (intraperitoneal injection) for 5 consecutive days followed by andrographolide (1, 10, or 20 mg/kg/day) for 12 weeks by intragastric gavage	↗ (heart)	-	-	-	-	SOD activity ↗; MDA and 4-HNE ↘; Nox2, Nox-4, p47^phox^, Nrf2, and HO-1 mRNA ↘	[54]
Balb/c mice	Toluene diisocyanate treatment (dermally and intranasally) for asthma induction with andrographolide treatment (0.1, 0.5, or 1 mg/kg, prophylatic regimen)	-	↗ (lung)	-	-	-	HO-1 protein ↗ (1 mg/kg, lung)	[97]
